# Genomic profiling of CHEK2*1100delC-mutated breast carcinomas

**DOI:** 10.1186/s12885-015-1880-y

**Published:** 2015-11-09

**Authors:** Maarten P. G. Massink, Irsan E. Kooi, John W. M. Martens, Quinten Waisfisz, Hanne Meijers-Heijboer

**Affiliations:** 1 Department of Clinical Genetics, VU University Medical Center, Amsterdam, The Netherlands; 2 Department of Medical Oncology, Erasmus MC Cancer Institute, Cancer Genomics Netherlands, Erasmus University Medical Center, Rotterdam, The Netherlands

**Keywords:** Breast carcinoma, *CHEK2*, Genomic profiling, Copy number aberration, Gene expression

## Abstract

**Background:**

CHEK2*1100delC is a moderate-risk breast cancer susceptibility allele with a high prevalence in the Netherlands. We performed copy number and gene expression profiling to investigate whether CHEK2*1100delC breast cancers harbor characteristic genomic aberrations, as seen for *BRCA1* mutated breast cancers.

**Methods:**

We performed high-resolution SNP array and gene expression profiling of 120 familial breast carcinomas selected from a larger cohort of 155 familial breast tumors, including *BRCA1*, *BRCA2*, and *CHEK2* mutant tumors. Gene expression analyses based on a mRNA immune signature was used to identify samples with relative low amounts of tumor infiltrating lymphocytes (TILs), which were previously found to disturb tumor copy number and LOH (loss of heterozygosity) profiling. We specifically compared the genomic and gene expression profiles of CHEK2*1100delC breast cancers (*n* = 14) with BRCAX (familial non-*BRCA1*/*BRCA2*/CHEK2*1100delC mutated) breast cancers (*n* = 34) of the luminal intrinsic subtypes for which both SNP-array and gene expression data is available.

**Results:**

High amounts of TILs were found in a relatively small number of luminal breast cancers as compared to breast cancers of the basal-like subtype. As expected, these samples mostly have very few copy number aberrations and no detectable regions of LOH. By unsupervised hierarchical clustering of copy number data we observed a great degree of heterogeneity amongst the CHEK2*1100delC breast cancers, comparable to the BRCAX breast cancers. Furthermore, copy number aberrations were mostly seen at low frequencies in both the CHEK2*1100delC and BRCAX group of breast cancers. However, supervised class comparison identified copy number loss of chromosomal arm 1p to be associated with CHEK2*1100delC status.

**Conclusions:**

In conclusion, in contrast to basal-like *BRCA1* mutated breast cancers, no apparent specific somatic copy number aberration (CNA) profile for CHEK2*1100delC breast cancers was found*.* With the possible exception of copy number loss of chromosomal arm 1p in a subset of tumors, which might be involved in *CHEK2* tumorigenesis. This difference in CNAs profiles might be explained by the need for *BRCA1*-deficient tumor cells to acquire survival factors, by for example specific copy number aberrations, to expand. Such factors may not be needed for breast tumors with a defect in a non-essential gene such as *CHEK2*.

**Electronic supplementary material:**

The online version of this article (doi:10.1186/s12885-015-1880-y) contains supplementary material, which is available to authorized users.

## Background

Approximately 10–15 % percent of all breast cancer cases arise within a familial clustering of multiple breast cancer cases. Inherited germ-line mutations in the high risk genes *BRCA1, BRCA2,* and *PALB2* are identified in approximately 20 percent of these breast cancer families. In addition, mutations in the *CHEK2*, *ATM* and *BRIP1* genes confer a moderate lifetime risk of breast cancer but are rare and account for less than 5 % of familial breast cancer cases [1, 2].

CHEK2*1100delC is a moderate-risk breast cancer susceptibility allele with a relatively high prevalence in the Netherlands of 1.1 % in the general population, 2.5 % in unselected breast cancer cases, and 4.9 % in familial breast cancer cases. Other mutations in the *CHEK2* gene contributing to breast cancer risk are negligible in the Dutch population. The lifetime risk of breast cancer for a female CHEK2*1100delC mutation carrier from the general population is 20–25 %, increasing to 35–45 % in a familial breast cancer setting [3–5].

*CHEK2* (*Checkpoint kinase 2*) has been shown to be involved in cell cycle control and DNA damage response. ATM (*Ataxia telangiectasia mutated*) phosphorylates CHEK2 in response to DNA damage, resulting in CHEK2 homodimerization. The resulting active kinase exerts its function through its ability to phosphorylate TP53, CDC25A, CDC25C, PLK and BRCA1 [6]. The function of *CHEK2* is abrogated by the 1100*delC frameshift mutation which causes a premature translation stop in the kinase domain of the protein. Both the mRNA, which is degraded through nonsense-mediated mRNA decay, as well as the resulting truncated protein are highly unstable [7, 8]. Very few breast tumors from 1100delC carriers show CHEK2 protein expression although LOH of the wild-type allele is infrequently found [9]. In contrast, LOH of the *BRCA1* gene is frequently reported in *BRCA1*-mutated breast cancers [10]. Also, *BRCA1* mutated breast tumors are frequently reported to be of the basal-like intrinsic subtype, opposed to breast tumors from *CHEK2* mutation carriers, which are reported to be mostly steroid hormone receptor positive (progesterone receptor/progesterone receptor (ER/PR) positive) [11]. In accordance, gene expression profiling assigns tumors from *CHEK2* mutation carriers to the luminal intrinsic subtypes [12].

We and others have shown specific somatic profiles of CNAs characteristic for both *BRCA1* and *BRCA2*-associated breast carcinomas [13–17]. These CNAs are thought to reflect specific oncogenic pathways in tumor development. The identification of driver genes in these genomic regions could lead to a better understanding of the underlying process of tumorigenesis and may provide novel clues for targeted therapies.

In this study we have performed high-resolution copy number, LOH and gene expression profiling of 120 familial breast carcinomas selected from a larger cohort of 155 familial breast tumors, including *BRCA1*, *BRCA2* and *CHEK2* mutant tumors. Samples were selected for low amounts of tumor infiltrating lymphocytes (TILs) by mRNA profiling because TILs have detrimental effects on genomic profiling of tumor material [18]. To ascertain whether CHEK2*1100delC breast cancers harbor characteristic genomic aberrations, as seen in *BRCA1* mutated breast cancers, we specifically compared the genomic profiles of 14 CHEK2*1100delC breast cancers and 34 BRCAX breast cancers of the luminal intrinsic subtypes for which both SNP-array and gene expression data is available. We compared our results with previously reported findings on genomic and gene expression profiling of CHEK2*1100delC breast cancers [19].

## Methods

### Ethics statement

This study has been approved by the medical ethical committee at Erasmus MC, and was performed according the Code of Conduct of the Federation of Medical Scientific Societies in The Netherlands. Anonymous or coded use of redundant tissue for research purposes is part of the standard treatment agreement with patients in our hospitals, and informed consent was therefore not required [20].

### Sample collection

Fresh-frozen specimens of primary breast tumors from female familial breast cancer cases were selected from the tissue bank of the Erasmus Medical Center Rotterdam. All cases had been screened for germ line mutations in *BRCA1*, *BRCA2* and for the CHEK2*1100delC mutation. The complete breast cancer cohort consists of 155 primary tumors and includes 26 tumors with a CHEK2*1100delC mutation, 47 *BRCA1*-mutated tumors, 6 *BRCA2*-mutated tumors, and 76 BRCAX tumors. These BRCAX breast cancer cases all originated from families with at least two breast cancer cases in first or second degree relatives of which at least one had been diagnosed before the age of 60. The entire cohort has been described in detail [12, 18]. In this study, 120 tumor samples for which both SNP array and gene expression data is available were used for further analyses. The gene expression and SNP microarray data have been deposited in NCBI's Gene Expression Omnibus and are accessible through GEO Series accession number 54219.

### Gene expression microarrays

For gene expression analysis. CEL files of the individual samples as deposited in GEO 54219 were used. The data was analyzed in Partek Genomics Suite (v6.6, Partek Inc.). Detection of differential gene expression was performed by ANOVA analysis, genes with FDR-stepup (false discovery rate) *p*-values less than 0.05 were considered to be statistically significant differentially expressed.

### Classification of intrinsic molecular subtypes

The intrinsic gene list was used to appoint the samples to molecular subtypes as described [12]. In short, the intrinsic gene list [21] was mapped to the corresponding probe-sets on the HGU_133_plus_2.0 array using Unigene Cluster Id's. The most variable probe-sets were used to cluster 120 familial samples using average linkage hierarchical clustering with correlation as a distance metric. In this paper, the luminal A and B samples together are referred to as luminal samples.

### mRNA based sample selection

To select for samples with relative low number of TILs, hierarchical clustering of expression data was used. This approach has largely been described in our previous work [18]. In short, the proportion of lymphocytic nuclei of 96 tumor samples was assessed on H&E-stained frozen sections. For subsequent mRNA analysis, the luminal and basal samples were processed separately. These samples were divided in two groups based on the TIL percentages (high and low TIL count, median split) on which subsequent ANOVA analysis was performed to find differentially expressed probe sets passing a FDR *p*-value <0.05. Finally, the overlapping probe-sets for the luminal and basal sample sets were determined to create the final mRNA immune signature, see Additional File 1. Following this approach, 14 out of a total of 17 CHEK2* 1100delC and 34 out of a total of 49 BRCAX breast cancers with relative low levels of this expression signature were selected for further supervised analyses.

### Copy number analyses by SNP arrays

For copy number and LOH analyses, CEL files of the individual samples as deposited in GEO 54219 were used. The array intensity. CEL files were processed by Partek Genomics Suite using default settings for background correction and summarization, results were corrected for GC-content and fragment length. Unpaired copy number analysis was performed in Partek Genomics Suite, comparing signal Log2 ratios to a custom created reference baseline of 90 female HapMap samples with European ancestry (CEU). The genomic segmentation algorithm was used to detect breakpoint regions and estimate copy number levels with stringent parameters (*P* < 0.0001, >20 markers, signal/noise: 0.45). With an expected normal range of 2 ± 0.25 copies. Differences between the tumor groups (mutation class) for frequency of copy number aberrations (gained, lost, or unchanged) were calculated by employing a 3 x 2 Fisher’s exact test (FE). Resulting *p*-values were not directly corrected for multiple testing. SNP array, gene and cytogenetic band locations are based on the hg19 Genome build. For unsupervised hierarchical clustering of copy number data the called copy number states (amplification (copy number >6), gain, loss or neutral) of the segmentation data were used as distance metric. Agglomerative clustering was performed on these data by Euclidean distance and Wards method.

### LOH calling by detection of allelic imbalance

A segmentation based approach of allelic imbalances was used to identify regions of LOH. The B-allele frequencies of the breast tumor samples were generated in Partek Genomics Suite. Mirrored B-allele frequency (mBAF) profiles were used as previously described [22]. The resulting mBAF profiles were segmented in Partek Genomics Suite and LOH calling of segmented regions was done by applying a fixed allelic imbalance threshold of 0.76 and *p*-value <0.01. The same parameters used in segmentation of the copy number data were applied, except that a window size of 100 SNPs instead of 20 SNPs was used as a minimum number of genomic markers.

## Results

### Sample selection for low amount of TILs

**Fig. 1:**
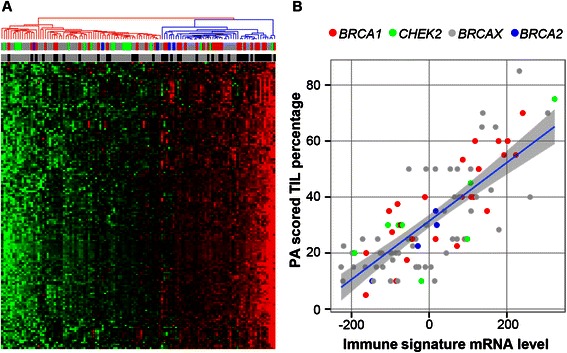
Hierarchical clustering of immune signature mRNA data and correlation with TILs. **a**, hierarchical clustering of mean centered, standardized immune signature gene expression data. Samples in the blue branch are regarded as high TIL (red: relative high expression, green: relative low expression), and are discarded from further analyses. Top row indicates mutation status (red: *BRCA1*, blue: *BRCA2*, green: *CHEK2*, grey: BRCAX), bottom row indicates intrinsic subtype (grey: luminal, black: basal). **b**, correlation plot for immune signature mRNA values and TIL percentages as determined by an experienced pathologist (r_s_ = 0.74, *p*-value < 0.001), colors represent mutation status

To select for samples with low number of TILs, hierarchical clustering of expression data of all 120 breast carcinomas based on the mRNA immune signature was used (Fig. 1a). Relative high numbers of TILs were predominantly found in basal-like breast cancers. However, 14 BRCAX and three CHEK2*1100delC samples of the luminal breast cancers were also found to have such high mRNA signature values and were not used in supervised class comparison of CNAs and differential gene expression analysis. Fig. 1b shows the correlation between immune signature mRNA values and TIL percentages as determined on H&E stained slides (r_s_ = 0.74, *p*-value < 0.001).

### Copy number and LOH profiling

High resolution copy number and LOH profiling by means of SNP array analysis was performed to gain insight into the genomic characteristics of CHEK2*1100delC as compared to BRCAX breast cancers. Intrinsic sub-typing of breast carcinomas based on global gene expression profiles has revealed large differences between the basal-like, ERBB2/Her2Neu and luminal subtypes regarding patterns of CNA's [13, 23–25]. As CHEK2*1100delC breast cancers are found to be exclusively of the luminal subtypes [12], the analyses were restricted to these intrinsic subtypes to avoid subtype associated confounding effects on copy number profiling.

**Fig. 2:**
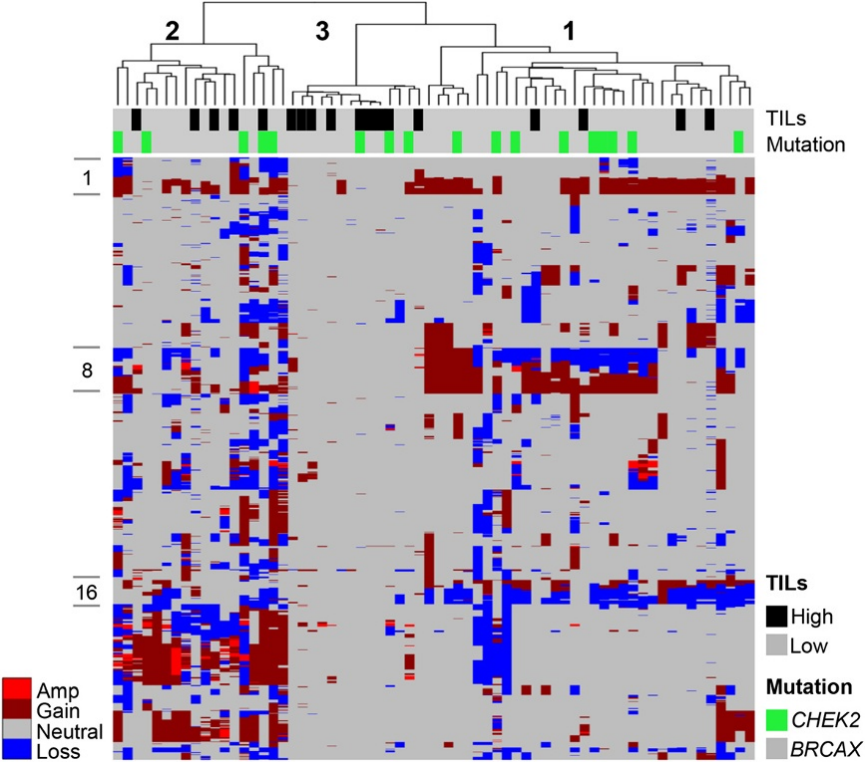
Genomic profiles of CHEK2*1100delC and BRCAX breast tumors. Hierarchical clustering of CNA data. On the vertical axis, chromosomes 1 to X are displayed. Copy number gains are indicated in dark red (copy number amplifications (copy number > 6) in light red), losses in blue, and copy neutral regions in grey. TIL (black: high TIL, grey: low TIL) and mutation status (green: CHEK2*1100delC, grey: BRCAX) are indicated for each sample in the top rows by color

Unsupervised hierarchical clustering of copy number profiles of all luminal CHEK2*1100delC and BRCAX samples, i.e. including samples with high TILs, suggests a great degree of heterogeneity amongst the CHEK2*1100delC breast cancers comparable to that seen in the BRCAX breast cancers (Fig. 2). The copy number aberration clustering roughly divides the samples into three groups, as indicated in Fig. 2. In group 1 many tumors are seen to have similar CNAs, including regions of copy number gain of chromosomal arms 1q, 8q, and 16p and copy number losses of chromosomal arms 8p and 16q. A second group (group 2) of tumors was found to have a more unstable CNA profile with focal amplifications on chromosome 17 (*ERBB2*) and concomitant high gene expression values for *ERBB2* and nearby genes (*GRB7, STARD3*), while a third group (group 3) is characterized predominantly by high TIL samples with very few CNAs. In agreement with the copy number analysis results, heterogeneous patterns of LOH with no frequent reoccurring regions were identified (data not shown).

**Fig. 3:**
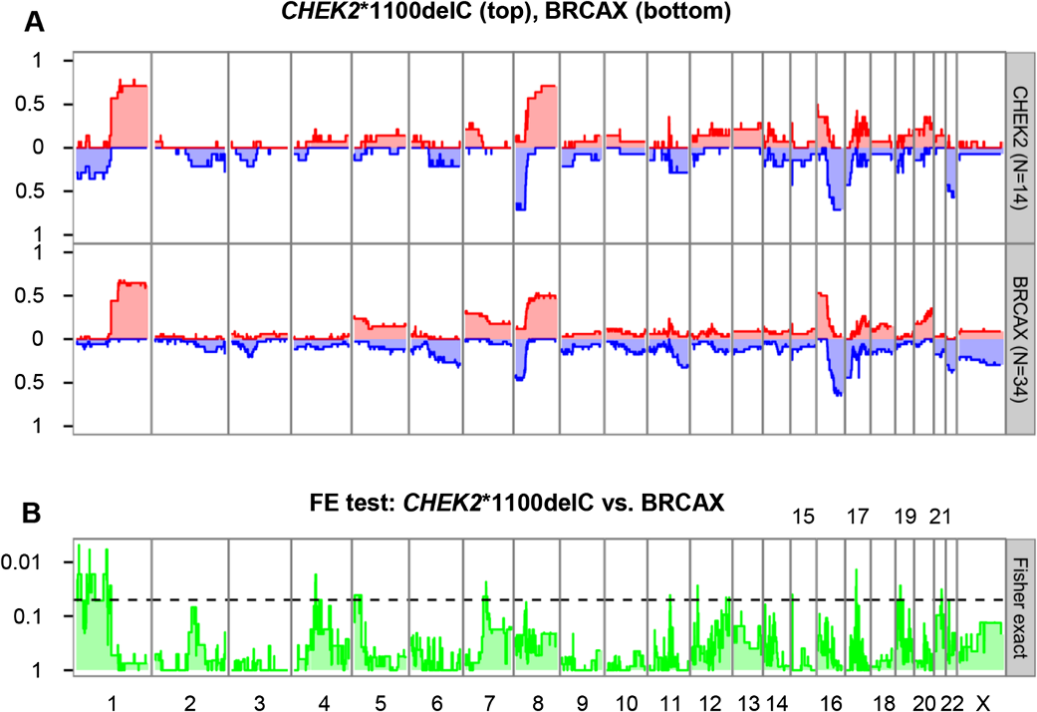
Copy number frequency plots of 14 CHEK2*1100delC and 34 BRCAX low TIL breast tumors. **a**, the frequency (x-axis) of gains (red) and losses (blue) are displayed along chromosomes 1 to X (y-axis) for 14 CHEK2*1100delC (top panel) and 34 *BRCAX* (bottom panel) breast cancers. **b**, Fisher's exact test is used to determine regions of differential copy number aberrations between the CHEK2*1100delC and *BRCAX* breast cancers. The dotted line represents a *p*-value threshold of 0.05 (not corrected for multiple testing). The regions above the threshold are considered to be significantly different between the groups. *P*-values are - log 10 transformed

Supervised class comparison of the copy number profiles of low TIL CHEK2*1100delC (*n* = 14) and BRCAX (*n* = 34) breast cancers identified a small number of genomic regions with differential CNAs (Fig. 3). Most notable is the copy number loss of chromosome 1p which overlaps with a previously reported region [19]. However, most of the identified regions are marginally statistically significant and have CNAs at very low frequencies in the two tumor groups. Interestingly, a small region of (focal) copy number gain on chromosome 17 (including the *ERBB2* locus) is found in almost half (6/14) of the CHEK2*1100delC breast cancers.

Copy number losses on chromosome 22 (including the *CHEK2* locus) are found in half of the CHEK2*1100delC breast cancers, of which 4 showed LOH at the CHEK2 locus. However, copy number losses on chromosome 22 are also frequently found for the BRCAX breast cancers (25 % of the cases). Furthermore, samples with copy number losses on chromosome 22 were seen in all 3 groups as identified by unsupervised hierarchical clustering of copy number data.

### Gene expression analysis

Gene expression analysis by means of ANOVA was restricted to low TIL samples of the luminal intrinsic subtypes, with mutation status as single factor (14 CHEK2*1100delC vs. 34 BRCAX). This resulted in 6 differentially expressed probe-sets passing a step-up FDR *p*-value of 0.05 (See Table 1). None of the differentially expressed genes between the CHEK2*1100delC and BRCAX breast cancers were found to overlap with the *CHEK2* signature reported by Muranen et al. [19].

**Table 1:** Differentially expressed probe-sets between *CHEK2*^a^1100delC and BRCAX breast cancers

Gene Symbol	Probeset ID	Chromosomal Location	FDR Stepup *p*-value (*CHEK2* vs. BRCAX))	Fold-Change (*CHEK2* vs. BRCAX)
NCRNA00201	225786_at	chr1q44	0.00339396	2.4
CENPJ	223513_at	chr13q12.12	0.00339396	1.8
OGT	209240_at	chrXq13	0.0208235	1.5
PRPF4B	202127_at	chr6p25.2	0.0208235	2
PIKFYVE	213111_at	chr2q34	0.0208235	1.5
NFYB	218127_at	chr12q22-q23	0.0284964	1.58

Details for differentially expressed probe-sets between *CHEK2*^a^1100delC and BRCAX breast cancers

FDR (False Discovery Rate)

## Discussion

We performed high-resolution copy number, LOH and gene expression profiling of CHEK2*1100delC breast cancers to better understand the tumorigenesis to which the germ line *CHEK2* deficiency predisposes. Our analysis was restricted to breast cancers of the luminal intrinsic subtypes as CHEK2*1100delC breast cancers are found to be exclusively of these mRNA based subtypes. Furthermore, samples were selected for low numbers of TILs based on a mRNA immune signature.

The copy number profiles of CHEK2*1100delC breast carcinomas were found to be heterogeneous and largely resemble those of the BRCAX breast carcinomas. The largest group of tumors has characteristic copy number gains of chromosomal arms 1q, 8q and 16p and copy number losses of chromosomal arms 8p and 16q. These CNAs have frequently been reported for breast carcinomas of the luminal intrinsic subtypes [13, 23–25]. A second group was found to have a more unstable CNA profile and focal amplifications of the *ERBB2* genomic region. The observed focal amplifications of the *ERBB2* genomic region and the increase in gene expression levels of the genes herein suggest that a proportion of CHEK2*1100delC breast cancers could be related to the *ERBB2*/*Her2Neu* intrinsic subtype. A third group was found to be largely CNA devoid; most of these samples were identified to have high numbers of tumor infiltrating lymphocytes (TILs). In previous work we identified a similar CNA devoid group of (*BRCA1*-mutated) basal-like breast carcinomas, which proved to be caused by the presence of large numbers of TILs in these samples.

Compared to the *BRCA1* and *BRCA2* profiles reported in literature and our previous study, copy number aberrations are infrequently seen in CHEK2*1100delC breast cancers. The most frequently observed aberrations in CHEK2*1100delC breast cancers are those seen in many breast cancers of the luminal intrinsic subtypes. Also, hierarchical clustering showed a great degree of heterogeneity of copy number profiles amongst the CHEK2*1100delC breast cancers, while *BRCA1*-mutated breast cancers frequently co-cluster in hierarchical cluster analysis [13].

Few characteristic CNAs for CHEK2*1100delC breast cancers were found. In contrast to our *BRCA1* profiling results [18], most of these CNAs in CHEK2*1100delC breast cancers are seen at very low frequencies and are marginally statistically significant. With the exception of copy number loss of chromosomal arm 1p, which was found more frequently in CHEK2*1100delC breast cancers. Furthermore, the observed copy number amplifications overlapping the *ERBB2* gene in the CHEK2*1100delC samples fits well with the reported over-expression of the *ERBB2* gene in half of the CHEK2*1100delC homozygous cases [26].

For the most part, our findings are comparable to previously reported findings on the genomic characteristics of CHEK2*1100delC breast cancers by Muranen et al. [19]. This includes the copy number loss of chromosomal arm 1p seen in CHEK2*1100delC breast cancers, and LOH/loss at the *CHEK2* locus in only part of the tumors. In line with this, Suspitsin et al. concluded that tumor-specific loss of the wild-type allele is not characteristic for breast cancers arising in *CHEK2* mutation carriers as well as for other moderate risk genes. [27]. In contrast, the reported copy number gains of the *CHEK2* region in CHEK2*1100delC breast cancers were not observed in our data, we only observed normal copy number and copy number losses.

In addition, genome wide gene expression analysis identified a small number of genes to be differentially expressed between CHEK2*1100delC and BRCAX breast cancers, of which none overlap with the previously reported *CHEK2* gene expression signature by Muranen et al [19]. Furthermore, only 2 genes are seen to overlap with a reported 40-gene *CHEK2* signature found in the study by Nagel et al. [12]. This difference is most likely due to sample selection criteria. Where our analysis is restricted to low-TIL BRCAX and CHEK2*1100delC mutated breast cancers of the luminal subtypes, Nagel et al. performed their analysis on all hormone receptor positive breast cancers, including high-TIL and *BRCA1*/*BRCA2* mutated samples. Furthermore, we applied a stringent false discovery *p*_value cut-off of 0.05, opposed to a FDR *p*_value of 0.25 by Nagel et al. Also, there is no overlap in the *CHEK2* gene signatures reported by Muranen et al. and Nagel et al.

The most significant differentially expressed gene in the 40-gene *CHEK2* signature is the *CHEK2* gene itself. We found *CHEK2* gene expression to be particularly high in the high-TIL BRCAX samples, this could explain why, after sample selection, we do not find the *CHEK2* gene to be differentially expressed.

All differentially expressed genes in our analysis were found to be relatively higher expressed in the CHEK2*1100delC samples as compared to the BRCAX samples, but were not found in genomic regions of copy number gain in the CHEK2*1100delC samples. We found no direct links between these differentially expressed genes and *CHEK2* gene function, except for possibly the *CENPJ* gene. This gene encodes a protein that belongs to the centromere protein family. The protein plays a structural role in the maintenance of centrosome integrity and normal spindle morphology [28]. Recently, *CHEK2* has been reported to be involved in the regulation of proper mitotic spindle formation through phosphorylation of BRCA1, hereby ensuring chromosomal stability [29].

For *BRCA1* mutated breast cancers, specific CNAs are reported. Complete loss of *BRCA1* leads to severe proliferation defects in normal cells, proving lethal during embryonic development [30]. Therefore, it seems likely that *BRCA1*-mutated cells acquire survival factors that allow *BRCA1*-deficient tumor cells to expand. CNAs are well known mechanisms to acquire such factors. For instance, in previous work we identified a region of copy number loss on chromosome 15q in all *BRCA1*-mutated samples, which likely acts on the reported *BRCA1* associated loss of *53BP1* [31]. In contrast to *BRCA1*, *CHEK2* deficiency is not lethal as evidenced by CHEK2*1100delC homozygous carriers in the population and the viability of Chek2 knockout mice [32, 33]. Therefore, specific survival factors for *CHEK2* mutated cells are not required during tumorigenesis if the wild type allele for *CHEK2* is lost. However, it remains unclear to what extent loss of the wild type allele of *CHEK2* is necessary for tumorigenesis. Although CHEK2*1100delC homozygous female carriers are more susceptible to tumor development [26], analysis of tumors from CHEK2*1100delC heterozygous carriers show a heterogeneous pattern of LOH/loss at the *CHEK2* locus. It remains uncertain whether this reflects two different tumor groups, i.e. one with complete loss of wild type *CHEK2* and thereby *CHEK2* driven tumorigenesis and one without thereby representing sporadic tumors, or that loss of one *CHEK2* allele is sufficient to drive tumorigenesis. Furthermore, loss of the *CHEK2* wild type allele could be a non-driver event.

## Conclusions

In conclusion, in contrast to *BRCA1*/*2* breast cancers, no apparent predominant specific CNA profile nor robust gene expression profile for CHEK2*1100delC breast cancers was found*.* This could in part result from the small number of CHEK2*1100delC breast cancer samples used in this study. However, in our previous work, an even smaller number of *BRCA1*-mutated basal-like breast cancers proved sufficient to identify *BRCA1*-associated CNAs. Nevertheless, we cannot exclude the possibility that multiple different *CHEK2* specific profiles do exist. Larger sample sizes are needed to investigate this.

The results show no specific tumorigenic events regarding *CHEK2* tumorigenesis, except for copy number loss of chromosomal arm 1p. However, gene expression analysis did not hint towards any potential driver genes in this region. Further studies are needed to establish whether this loss is indeed associated with *CHEK2* breast tumors. Also, gene expression analysis identified a very small number of differentially expressed genes between the CHEK2*1100delC and BRCAX breast cancers, of which, except for possibly *CENPJ*, none seem to have a putative role in *CHEK2* related tumorigenesis based on what is known in literature. This small number of differentially expressed genes can also, in part, be due to the small amount of CHEK2*1100delC samples used in the analysis, and need to be validated.

Based on our previous work on *BRCA1* tumors and the current study on *CHEK2* tumors we postulate a model in which breast tumors with a defect in an essential gene such as *BRCA1* or *BRCA2*, result in copy number profiles that reflect both the tumor subtype and specific surviving factors while breast tumors with a defect in a non-essential gene such as *CHEK2*, result in copy number profiles that largely reflect the tumor subtype. In this model the presence of a germ line CHEK2*1100delC mutation might be regarded as an accelerator of tumorigenesis leading to CNA profiles comparable to that of luminal sporadic breast tumors.

## Additional file

Additional file 1: **Immune signature probe-sets.** Excel spreadsheet with a list of probe-sets constituting the immune signature. (XLSX 18 kb)

## Abbreviations

TILs: Tumor infiltrating lymphocytes
LOH: Loss of heterozygosity
BRCAX: Familial non-*BRCA1*/*BRCA2*/*CHEK2**1100delC mutated
CNA: Copy number aberration
ER/PR: Estrogen/progesterone receptor
FDR: False discovery rate
mBAF: Mirrored BAF profiles
FE-test: Fisher’s exact test
